# Serratia marcescens Endocarditis

**DOI:** 10.7759/cureus.17346

**Published:** 2021-08-21

**Authors:** Sherif Elkattawy, Mahsa Mohammadian, Neil Williams, Ahmed Mowafy, Sarah Ayad, Muhammad Atif Masood Noori, Islam Younes, Kerry S Singh, Arthur Millman

**Affiliations:** 1 Internal Medicine, Rutgers-New Jersey Medical School/Trinitas Regional Medical Center, Elizabeth, USA; 2 Internal Medicine, Trinitas Regional Medical Center, Elizabeth, USA; 3 Internal Medicine, St. George's University, St. George, GRD; 4 Cardiology, Trinitas Regional Medical Center, Elizabeth, USA

**Keywords:** infective endocarditis, serratia marcescens, splenic infarcts, renal infarcts, intravenous drug use (ivdu).

## Abstract

Infective endocarditis (IE) secondary to *Staphylococcus aureus* and streptococcus species comprises the majority of cases in literature with Gram negative bacterial insults occurring infrequently. *Serratia marcescens* is a Gram negative bacillus which is classified as motile, non-lactose fermenting, and a facultative anerobe. The presumed risk factor for the development of *S. marcescens* IE is intravenous drug use (IVDU). We report two cases of IE causes by *S. marcescens*: first case describes IE of tricuspid and aortic valve requiring surgical intervention further complicated by epidural abscess. The second case was associated with renal and splenic infarct. These cases highlight the severity and complicated nature of *S. marcescens* IE. Given *S. marcescens* IE has been infrequently described in the literature, we believe that our cases are worth reporting to contribute to the present incidence and management of *S. marcescens* IE.

## Introduction

First described by William Osler in 1885, endocarditis is defined as inflammation of the inner layer of the heart, usually involving the heart valves and nearby supporting structures [1]. When the inflammation is secondary to an infectious agent such as bacteria, or less commonly fungi, the term "infective" endocarditis is used [2]. Initial presentations can be vague consisting of constitutional symptoms like fever, chills, arthralgia, anorexia, and weight loss. More sinister presentations are also not uncommon, unveiling their true nature through immunological and non-immunological manifestations seen in skin, eyes, and vital organs [3]. Large vegetations along endocardium and valves frequently embolize throughout the body with devastating complications. Intravenous drug use (IVDU) has widely been an associated risk factor for infective endocarditis (IE), most frequently affecting the tricuspid valve and to a lesser extent the mitral and aortic valves [4]. Infections secondary to *Staphylococcus aureus* and streptococcus species comprise the majority of cases in literature with Gram negative bacterial insults occurring infrequently. *Serratia marcescens*, a facultative Gram negative rod, is commonly associated with nosocomial infections and has only been implicated in the diagnosis of IE a handful of times [5]. In this case series, we will analyze occurrences of Serratia endocarditis secondary to IVDU with significant morbidity and mortality. 

## Case presentation

Case #1 

A 51-year-old male patient with past medical history of alcohol and IVDU, untreated hepatitis C, heart failure with reduced ejection fraction (left ventricular ejection fraction, LVEF 40%-45%), thoracic vertebral bacterial osteomyelitis, infective bacterial endocarditis with *S. marcescens*, s/p bioprosthetic aortic and tricuspid valves replacement was brought to the emergency department by emergency medical services (EMS) for evaluation of altered mental status, after being found unresponsive on the street. The patient had been previously admitted to the hospital a month prior to this presentation with similar complaints and was diagnosed with sepsis secondary to bacterial endocarditis and vertebral osteomyelitis with an epidural abscess. Blood cultures obtained on the prior admission grew Gram positive cocci in clusters, later identified as S. marcescens. The patient was treated with prolonged courses of antibiotics, after which his sepsis resolved, and he was discharged shortly after. Presumably, in the time between the two admissions, the patient had had valve replacement surgery performed, as he presented with a sternotomy scar and a trans-thoracic echocardiogram performed on this admission revealed bioprosthetic aortic and tricuspid valves as seen in Video 1. On the current presentation, the patient’s mentation was altered so no history could be gathered at the time of admission. On presentation, the patient was febrile, body temperature 101.8°F, blood pressure (BP) 11/64 mmHg, heart rate (HR) 110 bpm, respiratory rate (RR) 25/min, and his O2 saturation was 95% on room air. The complete blood count (CBC) revealed hemoglobin (Hgb) 11.2 g/dL (12-16 g/dL) with a mean corpuscular volume (MCV) of 77.9, white blood cell (WBC) count 3.6 (4.8-10.8) with absolute neutrophils of 2.8 (1.4-6.5) and platelet count 18k (130-400k).

**Video 1:** Trans thoracic echocardiogram revealing bioprosthetic aortic and tricuspid valve.

The basic metabolic panel (BMP) revealed blood glucose 99 mg/dL (74-118 mg/dL), creatinine 1.04 mg/dL (0.4-1.0 mg/dL), sodium 125 mmol/L (136-146 mmol/L), potassium 3.9 mmol/L (3.6-5.1 mmol/L), chloride 95 mmol/L (101-111 mmol/L), bicarbonate 21 mmol/L (22-32 mmol/L), magnesium 1.0 mg/dL (1.8-2.5 mg/dL), and phosphorus 1.6 mg/dL (2.4-4.7 mg/dL). Lactic acid was 1.2 mmol/L (0.5-2.2 mmol/L). Radiology was insignificant, as a chest X-ray revealed sternal sutures consistent with open heart and valve replacement surgery but showed no acute cardiopulmonary events. CT studies of the head and cervical spine were negative for acute, strokes, bleeds, or fractures as well.

The patient was bloused with 3 L of lactated Ringer in the ED, as code sepsis was called, and he was given STAT doses of vancomycin and zosyn for broad spectrum coverage. Blood and urine cultures were drawn for culture and sensitivity studies. The patient was started on standing doses of antibiotics and was admitted for further management. Unfortunately, the patient’s serum sodium dropped rapidly to 121 mmol/L, likely secondary to massive IV fluid transfusion, his serum osmolarity at the time was 260 mOsm/kg (280-295 mOsm/kg) and his urine osmolarity was 611 mOsm/kg; he was started on normal saline intravenous fluids (IVF), and was upgraded to the intensive care unit (ICU) for closer monitoring and correction of his electrolytes. The patient gradually regained consciousness, and his serum sodium improved to 127 in the span of eight hours, so the IV fluids were discontinued, and he was fluid restricted to prevent over-correction. Shortly after the patient became fully oriented, he opted to sign out of the hospital against medical advice. The risks were explained to the patient regarding the critical nature of his condition, but he decided to leave and not continue his hospital course.

A few hours later, the patient was again brought by the EMS after being found once again unconscious on the highway. His previous management was continued as mentioned before, and he regained consciousness shortly after admission. His condition improved in terms of body temperature, BP, and HR. Blood cultures obtained revealed Gram positive bacteremia, so his antibiotic regimen was narrowed to vancomycin alone. The patient then reported that he was on methadone treatment for heroine dependence and withdrawal and expressed the wish to be continued on it. A few hours later, the patient again wanted to leave the hospital against medical advice, and he was again counseled on the severity of his condition and the importance of completing his antibiotic regimen and hospital course, but he was adamant about leaving and left the hospital before the complete resolution of his symptoms.

Case #2

The patient was a 51-year-old Hispanic female with past medical history of hypertension, depression, chronic hepatitis C, and IV substance abuse who was brought to emergency room by ambulance with chief complaint of severe acute low back pain. Low back pain was started three days before her presentation. It was constant, radiating to legs, worsened with movement, with no alleviating factor, associated with malaise. The patient was actively using IV heroin and had previous history of heroin overdose. Her last heroin injection was two to three days before admission, around the same time that the pain started. Upon admission, the patient denied fevers, chills, chest pain, dyspnea, nausea, vomiting, diarrhea, skin lesions, numbness, tingling, and weakness of the lower extremities.

**Figure 1:**
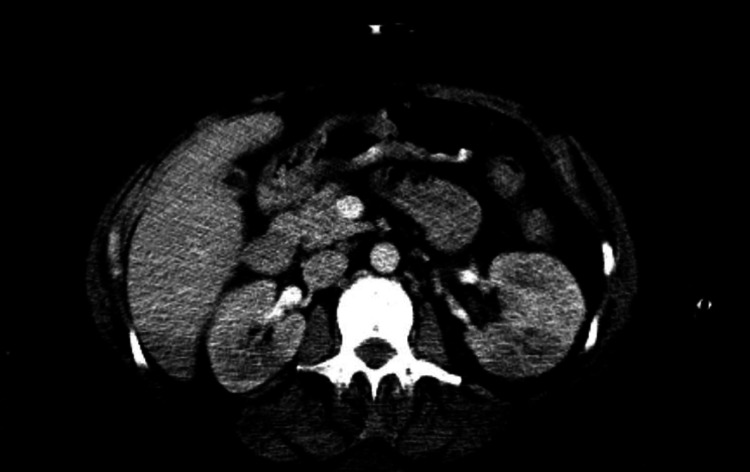
CT of the abdomen showing left and right renal infarcts.

**Figure 2:**
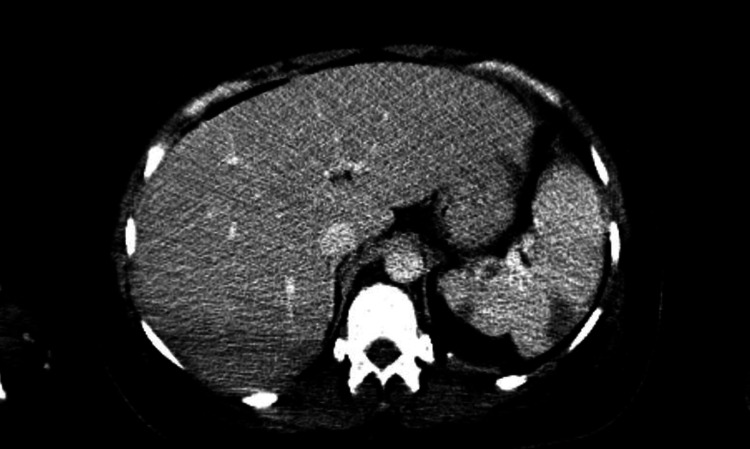
CT of the abdomen showing splenic infarcts.

In the ED, initial vital signs were within normal limits. Physical examination was remarkable for disorientation, 3/6 systolic murmur, in addition to opiate withdrawal sings. Labs were significant for sodium 129 (136-144 mmol/L), blood urea nitrogen (BUN) 30 (8-20 mg/dL), creatinine 1.5 (0.4-1.0 mg/dL), WBC 19.3 (4.8-10.8 k/uL) with neutrophil predominance, platelet 71 (130-400 K). Urinalysis was also positive for large blood and leuko esterase. MRI of spine showed degenerative changes but no concrete evidence of abscess or osteomyelitis. Trans thoracic echocardiogram was unremarkable. Trans esophageal echocardiogram showed moderate sized (1.6 cm x 2 cm) vegetation on the anterior leaflet of the mitral valve of high embolic potential and ischemic cardiomyopathy of the left ventricle as seen in Video 2. CT of the abdomen showed multiple splenic and renal infarcts as seen in Figures 1-2. The patient was started on empirical treatment with vancomycin and cefepime after first sets of blood culture was done. Blood cultures turned to be positive for *S. marcescens* and later antibiotics were changed to ceftriaxone based on the sensitivity results. 

**Video 2:** Trans esophageal echocardiogram showing moderate sized (1.6 cm x 2 cm) vegetation on the anterior leaflet of the mitral valve of high embolic potential and ischemic cardiomyopathy of the left ventricle.

During hospital course, the patient’s mental status was further deteriorated. Severe tachypnea and respiratory distress resulted in intubation and mechanical ventilation of the patient in the ICU. Brain MRI showed multiple foci of acute non-hemorrhagic infarcts secondary to cardioembolic disease. 

Antibiotic therapy was continued for the patient and second sets of blood culture were negative. The patient was evaluated by cardiothoracic surgeon. She was not a candidate for surgery intervention, due to unstable medical condition and multiple brain and myocardium infarcts. Hospitalization was further complicated by pneumonia with positive respiratory culture for *S. marcescens* and later *Clostridium difficile* infection. Due to multiple failed weaning trials, tracheostomy was placed for the patient. After two months of hospitalization, the patient was transferred to long-term acute care hospital. The patient was alert, able to follow simple commands, and remained ventilator dependent by the time of the discharge. 

## Discussion

*Serratia marcescens* is a Gram negative bacillus which is classified as motile, non-lactose fermenting and a facultative anerobe. The bacterium was first identified in 1819 by the Italian pharmacist, Bartomoleo Bizio, who isolated the organism after investigating reports of polenta developing a red pigment [5]. Unknown to the scientific community at the time, *S. marcescens* can be isolated from the soil, plants, animals, as well as water sources [5]. The pathogenicity of the organism remained largely unknown until recent decades when its association with IE and other diseases was first reported by Wheat et al. in 1951 [6-7]. Today, S. marcescens remains a rare cause of IE, with one study implicating the organism in 0.14% of IE cases [8]. As such, *S. marcescens* IE has been infrequently described in the literature, and there is still much to explore as it pertains to the pathogenesis, disease course, and management of this disease.

With regard to our patients, the presumed risk factor for the development of *S. marcescens* IE is IVDU. The distribution of reported cases in association with IVDU is geographically and temporally heterogeneous across the literature, although the overall frequency remains relatively low. Two landmark studies in 1976 and 1980 in San Francisco established a relationship between IVDU and the development of *S. marcescens* IE [9-10]. Of the 36 IE cases investigated, 32 patients (89%) endorsed a history of IVDU [6, 9-10]. Subsequent to these reports, however, the number of IVDU-associated Serratia IE cases plummeted to a total of two cases between 1980 and 2016 as noted by Phadke and colleagues [6, 11-12]. Of additional interest, no further reported cases arose in the San Francisco area within this period. Since *S. marcescens* can be found naturally in municipal water supplies [5], like Phadke and colleagues we hypothesize that the tap water utilized with the IVDU paraphernalia may be the most likely source of the *S. marcescens* inoculum encountered by our patients [6]. This may also lend an environmental explanation to the cluster of IVDU-associated *S. marcescens* cases encountered in San Francisco in the 1970s. 

Over the years, many studies have demonstrated that IVDU is associated with right-sided endocarditis [13]. However, the ubiquity of this notion has been challenged with the investigation of *S. marcescens* bacteremia as a confounder. Indeed, based on the preliminary San Francisco studies, 22 of the 36 patients (61%) had left-sided valvular involvement [9-10]. Furthermore, of the two IVDU-associated Serratia IE cases reported after 1980, both involved the aortic and/or mitral valves [6]. Intriguingly, the affinity of S. marcescens for left-sided valves holds true even in the absence of IVDU as a risk factor [6]. The mechanism for this predilection is unclear, but may involve the organism’s virulence factors, cardiac architecture, hemodynamics, endothelial surface protein expression, bacterial load, and composition of the injected drug [6, 13]. The affinity for left-sided valves, of course, aligns with the presentations of both of our patients. Patient 1 presumably had aortic valvulitis necessitating surgical intervention, while imaging of Patient 2 demonstrated a vegetation on the anterior leaflet of the mitral valve. 

Currently, there is no clear consensus on the optimal antibiotic regimen for the treatment of *S. marcescens* IE. Given the high mortality associated with this infection, Baddour and colleagues recommend surgical intervention 7-10 days following initiation of antibiotic therapy, but do not offer specific antibiotic recommendations for IE inflicted by *S. marcescens* [14]. The difficulty of selecting an appropriate regimen arises from the intrinsic resistance of *S. marcescens* to a myriad of antibiotics including penicillins, lower generation cephalosporins, macrolides, clindamycin, linezolid, tetracyclines, and rifampin [5-6]. Like several other members of the Enterobacteriaceae family, the genetic architecture of *S. marcescens* comprises the AmpC β-lactamase gene which is repressed at baseline [6, 15]. This is a potential weapon in the bacterium’s arsenal since the resultant β-lactamase can confer additional resistance to third generation cephalosporins and β-lactamase inhibitors [6, 15]. The expression of the AmpC β-lactamase gene can be upregulated by exposure to certain β-lactams and, theoretically, third generation cephalosporins [6, 16]. Consequently, there is presently some concern that clinical failure may occur following administration of a β-lactam despite an initial favorable sensitivity status, although it is uncertain if this is obtained with *S. marcescens* [16]. 

The therapeutic approach has been largely heterogeneous throughout the literature. Treatment regimens have included gentamicin, meropenem, levofloxacin, ceftriaxone, and piperacillin-tazobactam [6, 17]. Goodberlet et al. address this heterogeneity and the high mortality rate, reporting a favorable clinical outcome with meropenem 2 g every eight hours together with levofloxacin 750 mg every 24 h [17]. In the cases of our patients, the antibiotic regimen for Patient 1 is presently unknown, while Patient 2 was treated exclusively with ceftriaxone after sensitivities resulted. To the best of our knowledge, only one other study in the literature utilized ceftriaxone with a favorable result [6, 17]. It is possible that clinicians have become wary of the possibility of ceftriaxone to self-select for resistance, but we maintain that more studies need to be performed to determine the most effective antibiotic regimen. 

## Conclusions

*Serratia marcescens*, a facultative Gram negative rod, is commonly associated with nosocomial infections; however, it has been rarely linked to the diagnosis of IE a handful of times. In this case series, we will analyze occurrences of Serratia endocarditis secondary to IVDU with significant morbidity and mortality. In summary, we believe that our cases are worth reporting to contribute to the present medical knowledge of *S. marcescens* IE, and to ultimately help inform an appropriate treatment regimen.
